# Analysis of cell hyperplasia and parietal cell dysfunction induced by *Ostertagia ostertagi* infection

**DOI:** 10.1186/1297-9716-44-121

**Published:** 2013-12-11

**Authors:** Belgacem Mihi, Frederik Van Meulder, Manuela Rinaldi, Stefanie Van Coppernolle, Koen Chiers, Wim Van den Broeck, Bruno Goddeeris, Jozef Vercruysse, Edwin Claerebout, Peter Geldhof

**Affiliations:** 1 Department of Virology, Parasitology and Immunology, Faculty of Veterinary Medicine, Ghent University, Salisburylaan 133, 9820 Merelbeke, Belgium; 2 Department of Pathology, Bacteriology and Avian Diseases, Faculty of Veterinary Medicine, Ghent University, Salisburylaan 133, 9820 Merelbeke, Belgium; 3 Department of Morphology, Faculty of Veterinary Medicine, Ghent University, Salisburylaan 133, 9820 Merelbeke, Belgium

## Abstract

Infections in cattle with the gastric nematode *Ostertagia ostertagi* are associated with decreased acid secretion and profound physio-morphological changes of the gastric mucosa. The purpose of the current study was to investigate the mechanisms triggering these pathophysiological changes. *O. ostertagi* infection resulted in a marked cellular hyperplasia, which can be explained by increased transcriptional levels of signaling molecules related to the homeostasis of gastric epithelial cells such as *HES1, WNT5A, FGF10, HB-EGF, AREG, ADAM10* and *ADAM17*. Intriguingly, histological analysis indicated that the rapid rise in the gastric pH, observed following the emergence of adult worms, cannot be explained by a loss of parietal cells, as a decrease in the number of parietal cells was only observed following a long term infection of several weeks, but is likely to be caused by an inhibition of parietal cell activity. To investigate whether this inhibition is caused by a direct effect of the parasites, parietal cells were co-cultured with parasite Excretory/Secretory products (ESP) and subsequently analyzed for acid production. The results indicate that adult ESP inhibited acid secretion, whereas ESP from the L4 larval stages did not alter parietal cell function. In addition, our data show that the inhibition of parietal cell activity could be mediated by a marked upregulation of inflammatory factors, which are partly induced by adult ESP in abomasal epithelial cells. In conclusion, this study shows that the emergence of adult *O. ostertagi* worms is associated with marked cellular changes that can be partly triggered by the worm’s Excretory/secretory antigens.

## Introduction

Infections with the abomasal nematode *Ostertagia ostertagi* are considered as a major source of economic losses in cattle throughout the temperate regions of the world. *O. ostertagi* infection results in profound physio-morphological and functional alterations of gastric mucosal cells [1]. The gastric fundic mucosa is organized in well-defined units referred to as gastric glands composed by different cell lineages [2]. Homeostasis of this highly renewing epithelium is under a tight regulation of different molecular and cellular signaling pathways that keep a balance between proliferation and differentiation of the different gastric cell populations. Changes in the mucosal microenvironment induced by gastric infections lead to the disturbance of gastric cell homeostasis [3]. Abomasal ostertagiosis is characterized by mucous cell hyperplasia, impairment of parietal cell function and the replacement of functional parietal cells by an undifferentiated cell population [1,4]. The molecular mechanisms mediating these mucosal changes during an *O. ostertagi* infection remain largely unknown. Minor cellular changes are first confined around the nodules containing the immature larvae. After the emergence of adult worms from the gastric glands, the changes tend to become more general [5,6]. Huby et al. showed that the excretory/secretory products (ESP) of ruminant gastrointestinal nematodes could increase the proliferation of gastric cell lines [7]. In addition, Simpson et al. showed that the transplantation of *T. circumcincta* adult worms, confined in porous bags, lead to a significant increase of abomasal pH and serum gastrin within a few hours. Altogether, these data suggest a key role of ESP in the pathobiology of abomasal nematode infections [8]. Previous data showed that similar changes occur in response to bacterial, viral, and parasitic infections, suggesting the existence of a conserved host response [9-12]. It has been shown that these mucosal changes can be triggered by a local inflammatory response, as increased expression levels of pro-inflammatory factors such as IL1B, TNFA and prostaglandin E2 (PGE2) are associated with the impairment of parietal cell function and the alterations of mucosal cell homeostasis [13-16]. In addition to inflammatory factors, changes in expression levels of SHH (Sonic Hedgehog), FGF (Fibroblast Growth Factors), BMP (Bone Morphogenetic proteins), WNT (Wingless-Type) and NOTCH could induce an imbalance between cell proliferation and cell differentiation in the gastric mucosa [3]. The role played by all these factors in the pathogenesis of abomasal ostertagiosis is still unknown.

Therefore, in order to improve our understanding of the pathobiology of cattle ostertagiosis, the purpose of the current study was to investigate the pathophysiological alterations affecting mucosal cells and to unravel the changes in the signaling pathways that might generate these alterations. Finally, we also wanted to analyze whether the inhibition of parietal cell activity is triggered by a direct effect of *O. ostertagi* ESP and/or by increased levels of inflammatory factors.

## Materials and methods

### Infection trials, tissue collection and parasite material

The experimental design was described previously by Mihi et al. [17]. Briefly, nematode-free Holstein calves, aged 6 to 8 months, were randomly assigned into the different experimental groups. Three groups of four calves were orally infected with a single dose of 100 000 *O. ostertagi* L3 larvae/animal and killed after 6, 9 and 24 days post infection (dpi), respectively, corresponding to the presence of L3, L4 and adult stages. Another group of four calves was maintained uninfected and used as a negative control. For histological analysis, an additional group of three calves was infected with the same challenge and killed at 21 dpi. Furthermore, a group of four calves was maintained on a pasture to acquire a natural *O. ostertagi* infection and euthanized 60 days after the first exposure (60 days post exposure (dpe)). An additional group has been included in this study, in which six calves received 1000 L3 infective larvae per day during 30 days and were killed 60 days after the first challenge (60 dpi). The experimental protocol was carried out with the approval from the ethical committee of the Faculty of Veterinary Medicine at Ghent University. Parasite collection, culture and ESP purification were performed as previously described by Geldhof et al. [18]. The postmortem worm counts were performed according to the method described by Geldhof et al. [19]. LPS contamination of worms ESP was quantified using ToxinSensor TM chromogenic LAL endotoxin assay kit (GenScript) according to the manufacturer’s instructions.

### Cell culture

Bovine mucous epithelial cell culture was carried out following the method described by Hoorens et al. [20]. Isolation and culture of parietal cells was performed as previously described with some modifications [21]. In short, rabbit and bovine stomachs collected from slaughterhouses were washed and transported in cold PBS. The stomachs were inspected for the presence of inflammation or signs of infection. The mucus was removed using a glass slide and the mucosa was scraped off and minced in very small fragments. After successive washing steps with PBS and MEM (Invitrogen), the minced mucosa was digested for 30 min in MEM medium supplemented with 2 mg/mL collagenase Type 1 (Invitrogen) and 5 mg/mL BSA. The digestion was stopped by three-fold dilution of the collagenase solution and big undigested fragments were removed using a 220 μm mesh sieve. The gastric glands were allowed to settle down for 20 min after which the supernatant was discarded and the remaining glands were further dissociated mechanically by pipetting the solution up and down. The cells were washed and centrifuged three times at 350 *g* for 5 min. The resulting cells were filtrated through 40 μm cell strainers, washed three times in PBS and incubated in medium A containing: DMEM/F12 (Sigma Aldrich) supplemented with 20 mM Hepes, 0.2% BSA, 10 mM glucose, 8 nM EGF (Sigma Aldrich), 1× Insulin, Transferrin, Selenium Solution (ITS -G) (Gibco), 1% penicillin-streptomycin, 50 mg/mL amphotericin B and 25 μg/mL gentamycin (Invitrogen). The cells were washed in PBS and plated on chambered coverslips (Nunc) coated with Matrigel basement membrane (BD bioscience) diluted at 1/7 in ice-cold sterile water and incubated at 37 °C in medium A without amphotericin B.

### RNA extraction and cDNA synthesis

Total RNA was extracted from tissue samples using Trizol (Invitrogen) and further purified using the RNeasy Mini kit (Qiagen), while total RNA was extracted from cultured cells using the RNeasy Mini Kit (Qiagen) without previous Trizol treatment. Removal of contaminating genomic DNA was performed using DNase set (Qiagen) according to the manufacturer’s instructions. RNA quality was verified using an Experion™ Automated Electrophoresis System (Bio-Rad) and the concentrations were determined using a NanoDrop ND-1000 spectrophotometer (NanoDrop Technologies). Total RNA was converted into cDNA using the iScript cDNA synthesis kit (Bio-Rad), according to the manufacturer’s instructions.

### Quantitative Real-time PCR

Real time PCR reactions were carried out as described elsewhere [17]. Primer sequences are shown in Additional file 1. Amplification cycles were performed on a StepOnePlus Real-Time PCR System (Applied Biosystems) under the following conditions: 95 °C for 20 s followed by 35 cycles of 95 °C for 15 s, primer’s melting temperature (Additional file 1) for 15 s, and 72 °C for 15 s. A melting curve analysis was performed at the end of the reaction to ensure the specificity of the primers. The quantification of gene expression was carried out using the delta-delta CT method in cell culture experiments. The quantification of gene expression at the mucosal level following *O. ostertagi* infection was obtained by transforming the Ct values into relative quantities [17]. Gene expression was evaluated based on fold differences in gene transcription levels at different time points during the infection compared to the negative control animals. This was done by calculating the ratios of individual relative quantities on the geometric means of relative values of the control samples.

### Histology and immunofluorescence

For immunofluorescence, 5 μm formaldehyde-fixed tissue sections were successively deparaffinized in xylene, rehydrated in graded ethanol and rinsed in PBS. Sections were boiled in Antigen Retrieval Citra Microwave Solution and washed respectively 15 min in water and 5 min in PBS. Sections were permeabilized in 2% bovine serum albumin (BSA), 0.3% TritonX-100 PBS for 15 min, then blocked in 2% BSA in PBS for 45 min. Tissue sections were incubated with primary antibodies diluted 100 times (rabbit anti-ATP4A IgG (Calibiochem), rabbit anti-Ki-67 IgG (Abcam)) overnight at 4 °C. After washing with PBS, tissue specimens were incubated for 1 h with a secondary antibody diluted 100 times (Alexa Fluor 488 goat anti-rabbit IgG (Invitrogen)). DAPI (1.5 μg/mL) was used to counterstain the nuclei. Tissue sections were rehydrated in an ethanol gradient and were mounted in DEPX mounting medium (VWR). The slides were observed using a *LEICA* microscope. In order to count parietal cells, four random pictures were chosen from each stained section. Parietal cell count was performed using one ATP4A stained section per animal. Positive ATP4A stained cells were counted in a 200 μm wide area from the bottom of the glands to the mucosal surface using ImageJ software. The ATP4A staining of cultured parietal cells was carried out as described by Agnew et al. [21].

### Measurement of acid secretion

The method applied to measure acid secretion by parietal cells has been described previously by Mangeat et al. [22]. Briefly, parietal cells grown on Matrigel coated Lab-TeK chambered coverslips were pre-incubated during 15 min or 24 h with or without *O. ostertagi* ESP and/or with different secretagogues for 20 min. Parietal cells were then incubated for 10 min with 10^-6^ M of 9-aminoacridine (9-AA) (Excitation: 400 nm- Emission 450 nm). Cells were washed three times and perfused again with the same concentrations of ESP and/or secretagogue as used before. Ten random pictures were taken below the saturation levels of the 9-AA fluorescence. The fluorescence intensity and the area of 9-AA within the secretory vacuoles were quantified using ImageJ software. The mean fluorescence intensity and the area of fluorescent vacuoles were measured in the blue channel after subtraction of background fluorescence. The multiplication product of the mean fluorescence intensity and the area of acidic vacuoles were representative of acid secretion index per parietal cell.

### Statistical analysis

Statistical analysis was performed using GraphPad Prism software. For the infection trials, the data were analyzed using the Nonparametric Mann Whitney U test, by comparing the infected groups with the uninfected control. In cell culture experiments the analysis was carried out using the unpaired Student’s *t*-test by comparing each group of treated cells with the PBS control group. A *P-*value of ≤ 0.05 was considered as statistically significant.

## Results

### Postmortem worm counts

Postmortem worm counts (Additional file 2) showed that both natural and experimental *O. ostertagi* infections were successfully established. The numbers of worms recovered from naturally infected animals were substantially higher compared to the experimentally infected groups.

### Impact of *O. ostertagi* infection on mucosal cell proliferation

**Figure 1:**
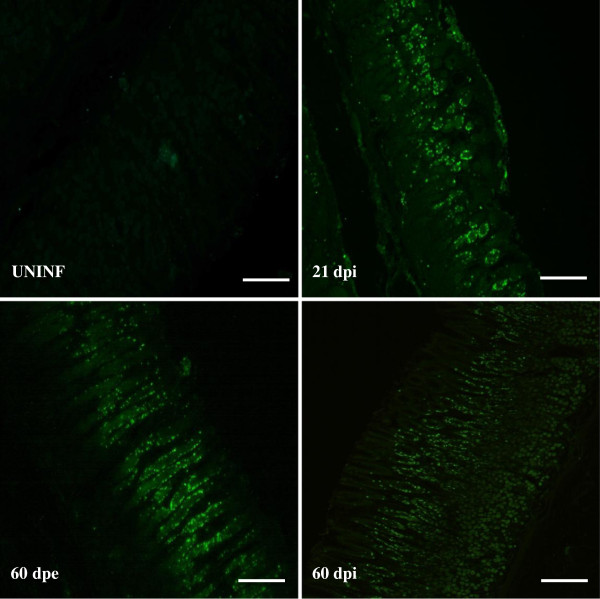
**Representative pictures of fundic sections stained for the proliferation marker Ki67.** Proliferative nuclei are stained in green. UNINF: uninfected, dpi: days post infection, dpe: days post exposure (Scale bar = 100 μm).

In order to determine the impact of an *O. ostertagi* infection on mucosal cell proliferation, tissue sections were stained for Ki-67 as a cell proliferation marker (Figure 1). A marked increase of stained nuclei located in the neck zone of all the gastric glands was observed following both single or trickle infection. One animal from the 21 dpi group only showed proliferation in areas surrounding the glands containing the larvae and did not show a significant increase of stained nuclei in non-invaded mucosa (data not shown)*.*

To determine which pathways are involved in the observed cellular hyperplasia and cell dedifferentiation, transcriptional changes of signaling molecules related to the homeostasis of gastric epithelial cells were investigated and the results are shown in Table 1. *WNT5A*, *HES1, AREG*, *HB-EGF a*nd *FGF10* mRNA levels were increased in all infected groups. *ADAM17* and *ADAM10*, two activators of several signaling molecules such as *HB-EGF*, were also significantly upregulated during both single and trickle infections. The highest increase observed for the impacted genes in single experimental infections was observed at 24 dpi.

**Table 1:** **Effect of an *O. ostertagi *** **on transcript levels of signaling network components involved in gastric cell homeostasis (mean fold difference compared to uninfected calves ± SEM)**

	**Single infection**			**Trickle infection**	
	**Experimental**			**Experimental**	**Natural**
**Gene**	**6 dpi**	**9 dpi**	**24 dpi**	**60 dpi**	**60 dpe**
**HB-EGF**	2.61 ± 1.3	4.47 ± 0.68*	7.12 ± 1.33*	6.45 ± 1.11**	5.13 ± 1.31*
**AREG**	3.57 ± 1.78*	4.01 ± 0.82*	11.40 ± 1.58*	7.79 ± 0.77**	8.86 ± 2.48*
**FGF10**	2 ± 0.2	2.34 ± 0.43	4.33 ± 0.45*	1.83 ± 0.13**	2.58 ± 0.46*
**HES1**	1.79 ± 0.24*	2.07 ± 0.34*	2.3 ± 0.3*	3.20 ± 0.36**	1.97 ± 0.2*
**ADAM10**	1.66 ± 0.83*	2.44 ± 0.6*	2.64 ± 0.68*	1.76 ± 0.19	2.14 ± 0.22*
**ADAM17**	1.96 ± 0.25*	2.16 ± 0.29*	3.5 ± 0.6*	2.41 ± 0.25**	2.66 ± 0.26*
**Wnt5A**	1.93 ± 0.17*	1.92 ± 0.31*	3.09 ± 0.22*	2.76 ± 0.30**	2.01 ± 0.31*
**SHH**	2.25 ± 0.68	2.26 ± 1.06	1.98 ± 0.49	2.93 ± 0.61**	1.81 ± 0.44
**BMP4**	1.31 ± 0.16	1.7 ± 0.36	1.63 ± 0.14	1.69 ± 0.15	1.26 ± 0.09
**FGF20**	2.27 ± 0.33	2.87 ± 1.78	1.95 ± 0.31	1.44 ± 0.26	2.94 ± 0.31*
**GIF**	1.7 ± 0.22	1.13 ± 0.05	0.85 ± 0.15	0.61 ± 0.01**	0.72 ± 0.15

*p value ≤ 0.05 uninfected animals vs infected animals.

**p value ≤ 0.01 uninfected animals vs infected animals.

### Effect of *O. ostertagi* infection on parietal cells and their activation

**Figure 2:**
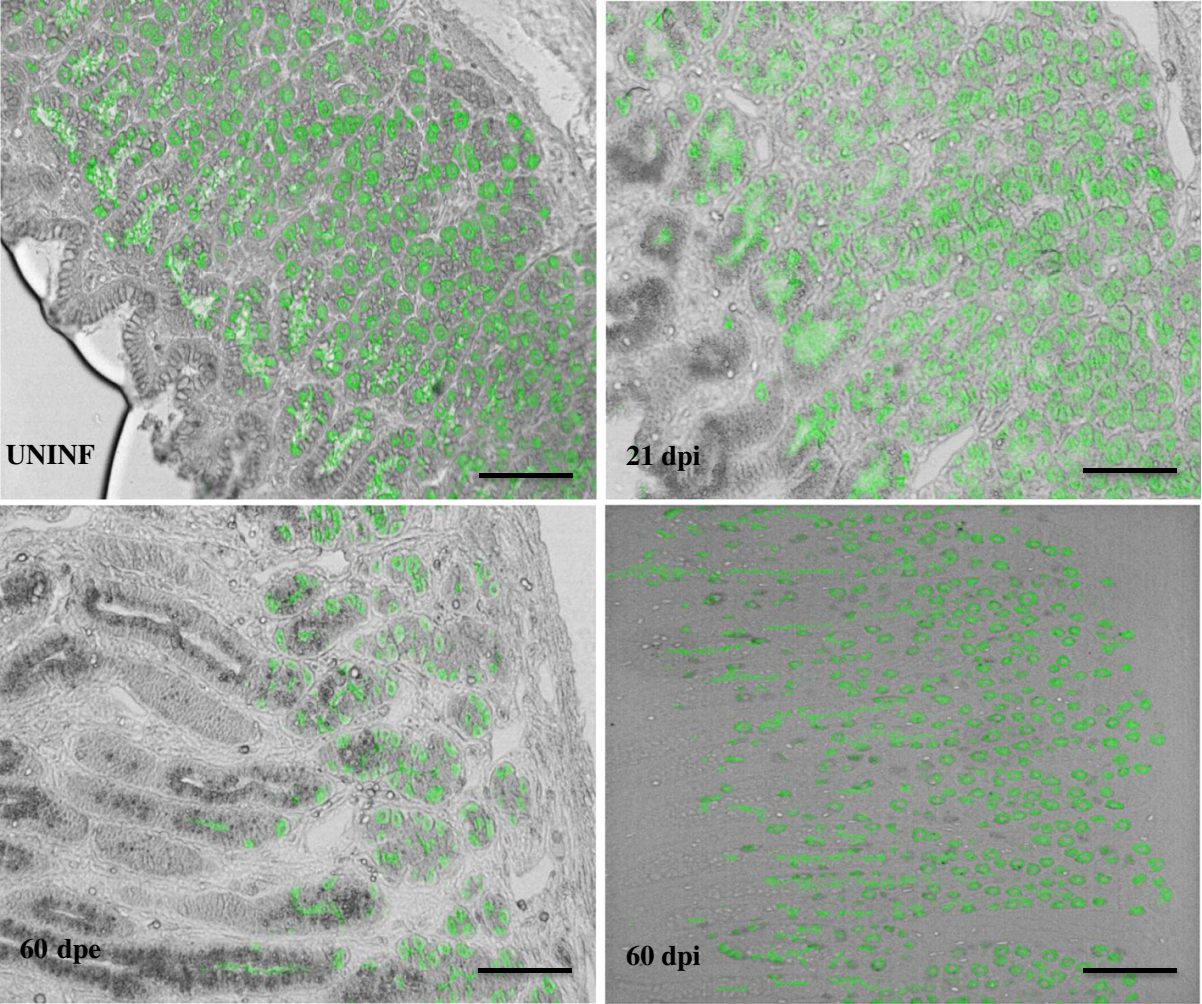
**Impact of an *O.ostertagi *infection on parietal cell numbers.** Parietal cells are visualized in fundic sections by immunostaining for ATP4A. Parietal cells are stained in green. UNINF: uninfected, dpi: days post infection, dpe: days post exposure (Scale bar = 100 μm).

It was shown previously that following *O. ostertagi* infection, there is an inhibition of gastric acid secretion [4]. In order to unravel the mechanism behind this inhibition, parietal cells were stained with ATP4A antibody and subsequently counted in the abomasal mucosa following an *O. ostertagi* infection. Although the total parietal cell number was unchanged at 21 dpi (128.33 ± 9.27) compared to the uninfected control animals (122.81 ± 5.5), a focal loss of parietal cells was consistently observed around gastric cysts, containing an immature larva. A significant diffused decrease of parietal cells was observed after both experimental and natural trickle infections (94.5 ± 4.27 and 62.31 ± 14.52) (Figure 2). In addition to the immunofluorescent staining, the transcriptional analysis of parietal cell markers shows that *ATP4A* and *AQP4* were significantly down regulated following a trickle infection, whereas *KCNQ1* transcript levels were unchanged in all animals (Table 2). All together, these data show that parietal cell loss occurs following a long period of parasite exposure.

**Table 2:** **Effect of an *O. ostertagi *** **on transcript levels of physiologic gastric acid secretion regulators (Mean fold difference compared to uninfected calves ± SEM)**

	**Single infection**			**Trickle infection**	
	**Experimental**			**Experimental**	**Natural**
**Gene**	**6 dpi**	**9 dpi**	**24 dpi**	**60 dpi**	**60 dpe**
**ATP4A**	1.62 ± 0.35	0.75 ± 0.08	0.49 ± 0.14	0.67 ± 0.04	0.29 ± 0.05*
**AQP4**	1.05 ± 0.53	0.99 ± 0.25	0.88 ± 0.08	0.64 ± 0.02**	0.42 ± 0.06*
**KCNQ1**	1.56 ± 0.17	1.11 ± 0.18	1.42 ± 0.24	1.27 ± 0.04	1.6 ± 0.25
**HDC**	1.57 ± 0.07*	1.33 ± 0.17	1.83 ± 0.4	0.94 ± 0.05	1.53 ± 0.37
**H2HR**	2.18 ± 0.23*	1.1 ± 0.24	1.34 ± 0.21	0.92 ± 0.09	0.69 ± 0.08
**CCKBR**	0.95 ± 0.22	1.39 ± 0.35	0.59 ± 0.15	0.70 ± 0.5*	0.72 ± 0.1

*p value ≤ 0.05 uninfected animals vs infected animals.

**p value ≤ 0.01 uninfected animals vs infected animals.

In order to understand how an *O. ostertagi* infection results in inhibition of gastric acid secretion upon the emergence of adult worms, in the absence of parietal cell loss, transcriptional levels of different genes involved in the endocrine activation process of parietal cells were assessed. The expression levels of the cholecystokinin B receptor (*CCKBR*) and histidine decarboxylase (*HDC*), which are implicated in histamine production by enterochromaffin like cells, were not impacted by the infection. Furthermore, the expression of the histamine receptor *HRH2*, which is responsible for parietal cell activation, was not affected by the infection, except on 6 dpi where a small increase was observed (Table 2).

### Impact of *O. ostertagi* ESP on parietal cell activity

**Figure 3:**
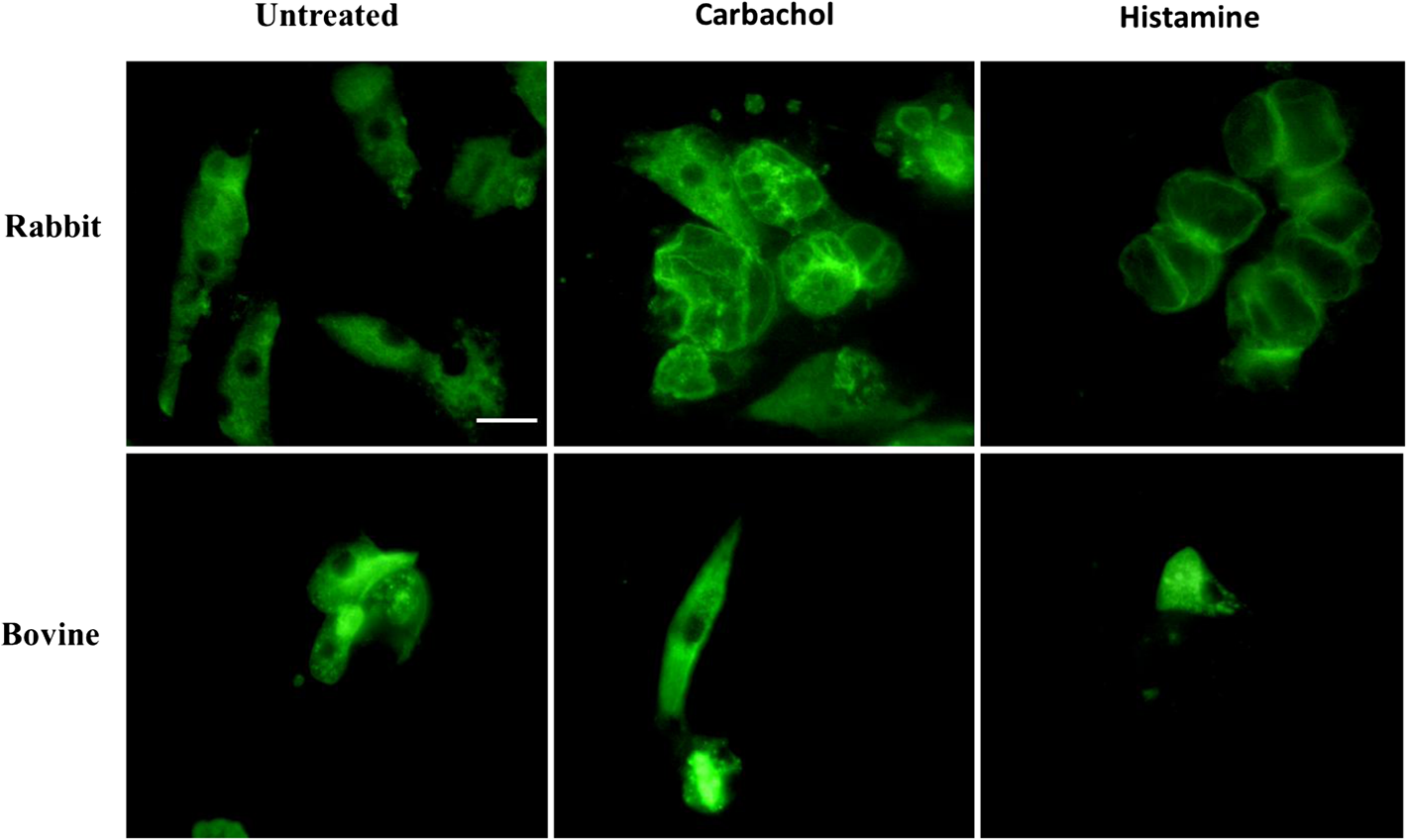
**Effect of histamine and carbachol treatment on ATP4A cytoplasmic distribution in cultured rabbit and bovine parietal cells.** Parietal cells were treated with histamine (10–5 M), carbachol (10–5 M) or PBS for 30 minutes prior to ATP4A staining. (Scale bar = 10 μm).

In an attempt to study the inhibitory activity of *O. ostertagi* ESP on the acid secretion of parietal cells, bovine parietal cells were first isolated and cultured. The proportion of attached bovine parietal cells obtained after 16 hours of culture was less than 5%. After 48 h, only a few bovine parietal cells were still attached to the coverslips. Under histamine and carbachol stimulation, bovine parietal cells did not show any reorganization of ATP4B location (Figure 3). These data suggest that the cultured bovine parietal cells were not responsive to the common secretagogues. In contrast, the same protocol applied on rabbit parietal cells resulted in 70% of attached parietal cells after being placed in culture for 16 h. In resting state, ATP4B staining was diffused in the cytoplasmic compartment, whereas under histamine and carbachol stimulation, ATP4B was translocated along secretory vacuoles (Figure 3). Therefore, the impact of *O. ostertagi* ESP on acid secretion was further investigated using cultured rabbit parietal cells.

**Figure 4:**
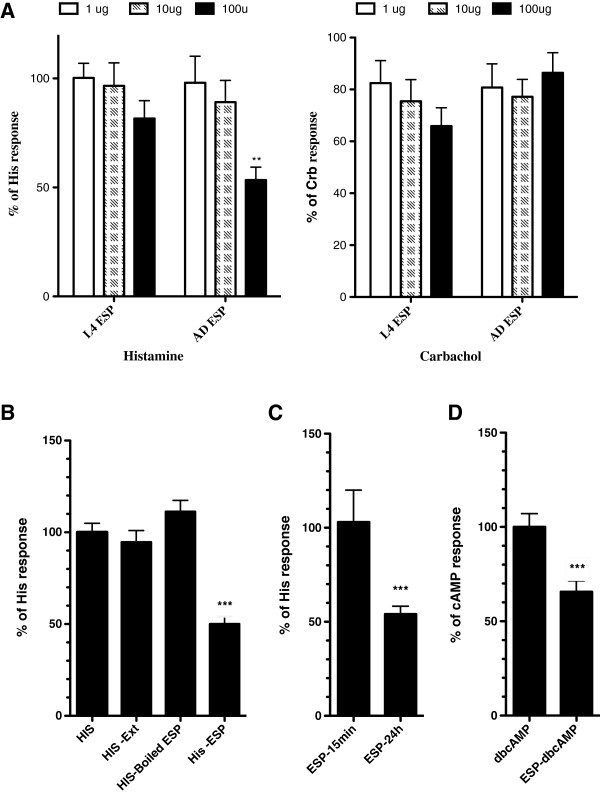
**Impact of *O. ostertagi *ESP on parietal cell activity. (A)** Cultured rabbit parietal cells were pre-incubated with parasite ESP at different concentrations for 24 h, and then stimulated with histamine (10–5 M) or carbachol (10–5 M) for 15 min. Quantitation of acid-secretion was measured using 9-aminoacridine accumulation as described in the material and methods. Data are expressed as percentages (means ± SEM) of secretory index in histamine or carbachol stimulated parietal cells in the absence of ESP treatment. **(B)** Quantification of acid secretion in histamine stimulated rabbit parietal cells following 24 h of pre-incubation with native adult extract, adult ESP and boiled ESP at 100 μg/mL **(C)** Quantification of acid secretion following 15 min and 24 h of incubation with adult ESP. **(D)** Quantification of acid secretion in dbcAMP stimulated rabbit parietal cells following 24 h of preincubation with 100 μg/mL adult ESP. Data are expressed as percentages (means ± SEM) of secretory index in dbcAMP or carbachol stimulated parietal cells in the absence of ESP treatment. (**p value ≤ 0.01, ***p value ≤ 0.001).

Cultured rabbit parietal cells were incubated for 24 h with different concentrations of adult or L4 ESP material. None of the L4 ESP concentrations showed any effect on histamine and carbachol stimulation (Figure 4A), whereas incubation of parietal cells with 100 μg/mL of adult ESP significantly inhibited the 9-AA accumulation by 53%. In contrast, no inhibitory effect was seen using the same concentration of adult ESP on carbachol stimulated cells. Furthermore, incubation of parietal cells with 100 μg/mL of adult worm protein extract or boiled adult worm ESP did not affect acid secretion (Figure 4B). In contrast with the 24 h pre-incubation period, 15 min pre-incubation of parietal cells with adult ESP at 100 μg/mL did not significantly alter the histamine induced acid secretion (Figure 4C). In order to investigate the impact of adult ESP on histamine receptor independent activation of acid secretion, parietal cells were stimulated with dbcAMP following 24 h of incubation with adult ESP. Adult ESP significantly reduced dbcAMP stimulation of acid secretion (Figure 4D).

### Mucosal inflammatory response induced by *O. ostertagi*

Previous studies showed that inflammatory factors could be involved in the impairment of gastric epithelial cell homeostasis and parietal cell function. Therefore, transcriptional changes of inflammatory related-genes were investigated in the abomasal mucosa following an *O. ostertagi* infection and the results are shown in Table 3. The inflammatory factors *IL1B, IL8* and *COX-2* exhibited increased transcriptional levels in the animals infected with a single dose of *O. ostertagi* compared to the uninfected group. In contrast, *TNFA* mRNA levels were not affected by the infection. The most important transcriptional changes following a single experimental infection occurred at 24 dpi. In addition, transcriptional analysis of inflammatory factors in animals receiving a trickle infection for a long period of time, shows that *IL-8* was significantly upregulated at 60 dpe and 60 dpi, whereas *COX-2, IL1B* and *TNFA* were only significantly upregulated in the 60 dpi group.

**Table 3:** **Effect of an *O. ostertagi *** **on transcript levels of inflammatory-related genes (Mean fold difference compared to uninfected calves ± SEM)**

	**Single infection**			**Trickle infection**	
	**Experimental**			**Experimental**	**Natural**
**Gene**	**6 dpi**	**9 dpi**	**24 dpi**	**60 dpi**	**60 dpe**
**IL1B**	3.01 ± 1.29	5.96 ± 0.84*	35.37 ± 10.77*	3.52 ± 0.49*	5.51 ± 1.86
**IL8**	3.11 ± 1.8	5.52 ± 1.6*	50.68 ± 14*	8.53 ± 1.55**	9.65 ± 2.66*
**TNFA**	1.81 ± 0.41	2.02 ± 0.31	5.44 ± 1.32	2.20 ± 0.0381*	3.76 ± 1.06
**COX-2**	1.05 ± 0.1	1.65 ± 0.2	6.45 ± 1.67*	2.92 ± 0.36**	1.69 ± 0.22

*p value ≤ 0.05 uninfected animals vs infected animals.

**p value ≤ 0.01 uninfected animals vs infected animals.

**Figure 5:**
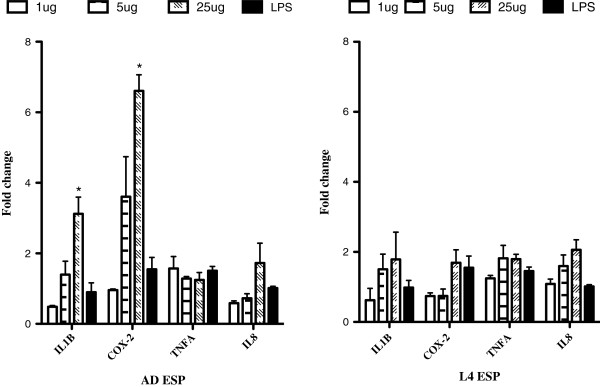
**Impact of *O. ostertagi *ESP on the transcription of inflammatory factors in bovine epithelial cells.** Relative expression levels of IL1B, IL8, TNFA and COX-2 were quantified in cultured bovine mucous epithelial cells following preincubation for 24 h with *O. ostertagi* adult and L4 ESP at different concentrations. In addition, cells were treated with 8 ug/mL LPS corresponding to the highest concentration of LPS contamination present in the ESP. Relative expression levels were normalized against RSP 29 and expressed as mean fold change ± SE. (*p value ≤ 0.05).

In order to investigate whether *O. ostertagi* ESP are implicated in the induction of these inflammatory factors, expression levels of *IL1B, COX-2*, IL8 and *TNFA* were measured in cultured bovine mucous cells treated with L4 and adult ESP for 24 h (Figure 5). Expression levels of *TNFA, COX-2, IL1B* and *IL8* were not impacted by L4 ESP treatment. In contrast, *IL1B* and *COX-2* mRNA levels were significantly higher in the cells treated with 25 ug/mL adult ESP, while *TNFA* and *IL8* transcriptional levels were unchanged by the same concentration compared to the PBS control.

## Discussion

In agreement with previous reports, our data show that *O. ostertagi* infection results in marked mucosal alterations affecting gastric epithelial cells. These changes are mainly associated with the development and appearance of adult worms in the abomasum.

Our data show that *O. ostertagi* infection triggers a prominent mucous cell hyperplasia in the neck zone of the fundic mucosa. Our data also show that the abomasal hyperplasia is associated with enhanced transcriptional levels of *HES1*, *WNT5A* and *FGF10*. These signaling molecules have been shown to play an important role in the maintenance of stem cell proliferation in the gastric isthmus [3]. It has been shown that the activation of WNT signaling in the gastric mucosa leads to an expansion of undifferentiated stem cells [23]. In addition, overexpression of *FGF10* in chicken embryos leads to increased levels of BrdU incorporation in the stomach, attesting to the presence of glandular cell proliferation [24]. Furthermore, it has been shown that *HES1* is present in proliferating cells within the isthmus of gastric glands [25]. The overexpression of *BHLH*, a repressor of *HES1,* leads to an excessive endocrinal cell differentiation in the developing stomach, suggesting that *HES1* plays a negative role on cell differentiation [26]. *IL8*, *HB-EGF*, *AREG, ADAM10 and ADAM 17*, which were all upregulated during *O. ostertagi* infection, may also have a trophic effect on abomasal epithelial cells. Joh et al. proposed a model where the activation of ADAM by IL8 can result in the cleavage of the HB-EGF precursor and the generation of an HB-EGF ligand, which in turn can induce epithelial cell proliferation [27].

It is also tempting to correlate the hyperplastic changes induced by *O. ostertagi* with the increased levels of inflammatory factors. It has been shown that IL1B stimulates gastric cell proliferation in vitro [28,29]. Furthermore, previous studies conducted in mice showed that overexpression of IL1B and PGE2, which is a metabolic product of COX-2, causes an increase of gastric epithelial cell proliferation [15,23]. On the contrary, it is also possible that an increase of gastrin secretion reported in several studies [30,31] could play a key role in triggering the observed hyperplastic changes by regulating the pathways discussed above. For example, it has been shown that INS-GAS transgenic mice overexpressing gastrin have a thickened hyperplastic mucosa [32].

In addition to the impact of gastrin on epithelial cell proliferation, the hypergastrinemia has also been associated with a marked decrease of parietal cell numbers in INS-GAS mice [32]. This might explain the parietal cell loss and subsequently the reduction of acid secretion after infection with *O. ostertagi* for a long period of time. Previous studies showed that ostertagiosis results in a marked reduction of abomasal acid production [4,33-35], associated with the emergence of adult worms from the fundic mucosa [4]. Several reports showed that a similar effect on acid secretion was observed only a few hours after the transplantation of adult *T. circumcincta* and *O. ostertagi* in the abomasum of naïve animals [34,36]. Furthermore, transplantation of *T. circumcincta* adult worms, which were prevented from mucosal contact, leads to reduced abomasal acid output also within a few hours [8]. Our study shows that the emergence of adult worms in the abomasum was not accompanied by a significant disappearance of parietal cells, suggesting that the rapid increase of the gastric pH, which is associated with the emergence of adult abomasal nematodes, is more likely due to the inhibition of parietal cell function rather than a reduction in parietal cell numbers.

Our data show that ESP produced by adult *O. ostertagi* could be implicated in the inhibition of gastric acid secretion. The incubation of parietal cells with adult ESP resulted in the inhibition of their activity. Furthermore, heat treatment of adult ESP resulted in the abolishment of the inhibitory effect, suggesting that the impairment of acid secretion is mediated by worm secreted/excreted protein requiring an adequate folding to mediate its effect. Previously, Merkelbach et al. showed that adult ESP of *H. contortus* inhibited acid production in histamine stimulated rabbit gastric glands. The inhibitory effect was attributed to the presence of ammonia in adult ESP [37]. However, the authors did not investigate the impact of high molecular weight products on acid secretion. Our data also show that exposure of parietal cells to adult ESP for a short period of time did not impair the histaminergic stimulation of parietal cells. A direct inactivation of the histamine receptor with *O. ostertagi* ESP through a competitive antagonism would have resulted in a rapid inhibition of acid secretion. Therefore, the observed inhibitory effect of adult ESP is probably not mediated by a direct interaction of ESP with the histamine receptor. Previous studies showed that the activation of the histamine receptor in parietal cells results successively in increased levels of cytoplasmic cAMP, activation of protein kinase A (PKA) and the reorganization of the cytoskeleton, leading to the formation of secretory canaliculi. We show that adult ESP was able to reduce acid secretion in cAMP-stimulated parietal cells, suggesting that the inhibition of parietal cells by *O. ostertagi* ESP was not triggered at the cAMP generation level by the histamine receptor. Therefore, the molecular site of parietal cell inhibition by adult ESP should be at the level of the downstream effectors involved in the formation of secretory vacuoles.

The marked increase in *IL1B* and *COX-2* expression associated with the presence of the adult stage worms might also explain the rapid increase of abomasal pH. Our data show that adult ESP induced the production of IL1B and COX-2 in epithelial cells, suggesting that adult ESP might inhibit parietal cell activity by inducing inflammatory cytokine production in mucous epithelial cells. However, whether mucous epithelial cells in vivo are exposed to similar ESP concentrations as used in the in vitro experiment remains unknown. Previous reports showed that the inflammatory factors such as *IL1B*, *TNFA* and *PGE2* are able to alter parietal cell function [13,16]. *IL1B* and *TNFA* have been shown to inhibit both cholinergic and histaminergic stimulated rabbit parietal cells, while *PGE2* can inhibit histamine-activated parietal cells through the inhibition of cAMP generation by Adenylate Cyclase following histamine stimulation. Interestingly, our data show that the mechanism of parietal cell inhibition by adult ESP differs from those described above for the inflammatory factors, suggesting that both pathways can be active in the inhibition of parietal cell activity during an *O. ostertagi* infection.

In the present study we used rabbit parietal cells as a model to investigate the impact of *O. ostertagi* ESP on gastric acid secretion. This was motivated by the fact that, unlike cultured rabbit parietal cells, bovine parietal cells are not responsive to histamine and carbachol stimulations. Previous attempts to obtain responsive gastric glands from sheep and cows were also not successful [37,38]. Dispersed bovine gastric glands are insensitive to histamine, however they are stimulated by dbcAMP treatment [38]. In our hands, cultured bovine parietal cells did not show any morphological feature of activation following histamine and carbachol stimulation as well as dbcAMP treatment (data not shown). In the same manner Merkelbach et al. showed that sheep gastric glands were insensitive to histamine stimulation and subsequently they were not suitable to investigate the impact of the worm chemicals on acid production [37]. Despite the unresponsiveness of ruminant parietal cells in vitro to the common secretagogues, subcutaneous injection of histamine and carbachol induces a decrease in pH in sheep abomasum. Furthermore, the administration of cimetidine and atropine, which are respectively antagonists of histamine and carbachol, blocks the acid secretion in sheep [35]. This suggests that similarly to monogastric species, the acid secretion is mediated by histamine and carbachol stimulation in ruminants. The absence of a bovine parietal cell response in vitro might be due to the inadequacy of the isolation and culture protocol for ruminant species.

In conclusion, this study shows that adult *O. ostertagi* is responsible for marked mucosal cell hyperplasia associated with alteration of several signaling pathways involved in the maintenance of gastric epithelial cell homeostasis. Parietal cell numbers were only decreasing after prolonged infection but their function can be inhibited early during primary infection by adult worm ESP. Furthermore, our data show that the mucosal cell hyperplasia and the impairment of parietal cell function could be triggered by the upregulation of inflammatory factors which are in turn partly induced by adult ESP.

## Competing interests

The authors declare that they have no competing interests.

## Authors’ contributions

BM, MR, SVC, BG, JV, EC and PG conceived and designed the experiments. BM and FVM performed the experiments. WVB and KC participated in the staining work. BM and FVM analyzed the data and performed the statistical analysis. BM drafted the paper and EC, SVC and PG modified and refined it. All authors read and approved the final manuscript.

## Supplementary Material

Additional file 1: Table S1 List of primers used for qRT-PCR analysis. Additional file 1 presents the complete list of Genes used in the qRT-PCR assay, indicating GeneBank accession number, primer sequences and melting temperatures (Tm). Click here for file

Additional file 2: Table S2 Postmortem worm counts. Additional file 2 shows the infection doses given to the different experimental groups, the average worm counts recovered at necropsy from the abomasa and the percentage of adult worms. Click here for file
