# Supplementary material for: Analysis of cell hyperplasia and parietal cell dysfunction induced by Ostertagia ostertagi infection
Source: Vet Res. 2013 Dec 11;44(1):121. doi: 10.1186/1297-9716-44-121 (PMC3878833; doi:10.1186/1297-9716-44-121)
Supplement: Additional file 1: Table S1 — List of primers used for qRT-PCR analysis. Additional file 1 presents the complete list of Genes used in the qRT-PCR assay, indicating GeneBank accession number, primer sequences and melting temperatures (Tm). [file 1297-9716-44-121-S1.docx]

| **Gene Symbol** | **Accession number** | **Primer sequences** | **Tm** |
| --- | --- | --- | --- |
|  |  |  |  |
| ***ADAM10*** | NM_174496.2 | F: AAACATGGCTTGGAGGAGTG | 60 |
|  |  | R: CCAGGTTGCAGGGTGATAGT |  |
|  |  |  |  |
| ***ADAM17*** | XM_595713.4 | F: GCATTCTGGTCCATTGTGTG | 64 |
|  |  | R: ACGTTACTGGGGTGGAACAG |  |
|  |  |  |  |
| ***AQP4*** | NM_181003.2 | F: TAGGAGCTGTCCTTGCTGGT | 60 |
|  |  | R: CTGTTGTCCTCCACCTCCAT |  |
|  |  |  |  |
| ***ATP4A*** | NM_001144089.1 | F: GATGTGGAGGCCATGAACTT | 62 |
|  |  | R: TTGAGCACAGCATCAGGAAC |  |
|  |  |  |  |
| ***AREG*** | BC141281.1 | F: TGGTCA | 60 |
|  |  | R: GTCGATCACGGAGGACAGTT |  |
|  |  |  |  |
| ***BMP4*** | NM_001045877.**1** | F: GCTTCCACCACGAAGAACAT | 60 |
|  |  | R: TACGATGAAAGCCCTGATCC |  |
|  |  |  |  |
| ***CCKBR*** | NM_007627.4 | F: GGACAGACGGATTCCTACCA | 62 |
|  |  | R: GCCTCCTTTTCTGTGTGAGG |  |
|  |  |  |  |
| ***COX-2*** | NM_174445.2 | F: GCCAGGAAGTCTTTGGTCTG | 60 |
|  |  | R: TCTGGAACAACTGCTCATCG |  |
|  |  |  |  |
| ***FGF10*** | NM_001192165.**1** | F: ATCAGTGTGGCAGTGGGACT | 60 |
|  |  | R: GGCCAGTGTCTCCGTGTTTA |  |
|  |  |  |  |
| ***FGF20*** | NM_001192165.**1** | F: ATCAGTGTGGCAGTGGGACT | 60 |
|  |  | R: GGCCAGTGTCTCCGTGTTTA |  |
|  |  |  |  |
| ***GIF*** | NM_001206239.**1** | F: ACCTAGATGTGCCCCAAGTG | 60 |
|  |  | R: GTGATGTTGGAGGCTGAGGT |  |
|  |  |  |  |
| ***HDC*** | NM_001024551.**1** | F: GAAGAGGCTCCAGACGTCAC | 62 |
|  |  | R: GCCTTCCTCTTGTTCTGCAC |  |
|  |  |  |  |
| ***HB-EGF*** | NM_001144090.1 | F: CCCAGGTTATCACGGAGAGA | 60 |
|  |  | R: ATGAGAAGCCCCATGATGAC |  |
|  |  |  |  |
| ***HES1*** | NM_001034678.1 | F: CCAAAGACAGCATCTGAGCA | 60 |
|  |  | R: TTCCGGAGGTGTTTCACTGT |  |
|  |  |  |  |
| ***HRH2*** | XM_599517.2 | F: CATCACCCTGTCCTTCCTGT | 60 |
|  |  | R: TGACCTGGACTTTGCACTTG |  |
|  |  |  |  |
| ***IL1B*** | NM_174093.1 | F: AAGGCTCTCCACCTCCTCTC | 60 |
|  |  | R: TTTGGGGTCTACTTCCTCCA |  |
|  |  |  |  |
| ***IL8*** | NM_173925.2 | F: GTTGCTCTCTTGGCAGCTTT | 60 |
|  |  | R: GGTGGAAAGGTGTGGAATGT |  |
|  |  |  |  |
| ***KCNQ1*** | NM_001205441.1 | F: TACTGTCCACCATCGAGCAG | 60 |
|  |  | R: TACTCCGTCCCGAAGAACAC |  |
|  |  |  |  |
| ***SHH*** | XM_614193.3 | F: AGCAGTTTATCCCCAACGTG | 60 |
|  |  | R: TAAGGCGTTCAGCTTGTCCT |  |
|  |  |  |  |
| ***TNFA*** | NM_173966.2 | F: GCCCTCTGGTTCAGACACTC | 60 |
|  |  | R: AGATGAGGTAAAGCCCGTCA |  |
|  |  |  |  |
| ***WNT5A*** | NM_001205971.1 | F: CCTTCGCCCAGGTTGTAATA | 60 |
|  |  | R: CTGTCCTTGGGAAAGTCCTG |  |
|  |  |  |  |
