# Supplementary material for: Analysis of cell hyperplasia and parietal cell dysfunction induced by Ostertagia ostertagi infection
Source: Vet Res. 2013 Dec 11;44(1):121. doi: 10.1186/1297-9716-44-121 (PMC3878833; doi:10.1186/1297-9716-44-121)
Supplement: Additional file 2: Table S2 — Postmortem worm counts. Additional file 2 shows the infection doses given to the different experimental groups, the average worm counts recovered at necropsy from the abomasa and the percentage of adult worms. [file 1297-9716-44-121-S2.docx]

| **Groups** | **Challenge** | **Average Worm counts** | **Range** | **Percentage of adult worms** |
| --- | --- | --- | --- | --- |
| **Controls** | - | - | - | - |
| **6 dpi** | 10^5^ larvae on day 0 | 11717 | 8700-15700 | 0% |
| **9 dpi** | 10^5^ larvae on day 0 | 21255 | 9000-42000 | 0% |
| **24 dpi** | 10^5^ larvae on day 0 | NC | NC | NC |
| **60 dpi** | 10^3^ larvae/ day during 30 days | 8383 | 5700-10800 | 63.1% |
| **60 dpe** | Natural infection | 146659 | 81950-189700 | 21.4% |

NC Not Counted
